# Long non-coding RNAs: crucial regulators of gastrointestinal cancer cell proliferation

**DOI:** 10.1038/s41420-018-0051-8

**Published:** 2018-04-27

**Authors:** Jiaxin Chen, Shuiping Liu, Xiaotong Hu

**Affiliations:** Biomedical Research Center and Key Laboratory of Biotherapy of Zhejiang Province, Sir Run Run Shaw Hospital, Zhejiang University, Hangzhou, China

## Abstract

Studies of long non-coding RNAs (lncRNAs) have been prevalent in the field of non-coding RNA regulation in recent years. LncRNAs exert crucial effects on malignant cell processes in the gastrointestinal system, including proliferation. Aberrant lncRNA expression, through both oncogenes and tumor suppressor genes, is instrumental to tumor cell proliferation. Here, we summarize the different molecular mechanisms and relevant signaling pathways through which multifarious lncRNAs regulate cell proliferation and we show that lncRNAs are potential biomarkers for gastrointestinal cancers.

## Facts

- Proliferation of gastrointestinal cancers is a consequence of dysregulated signaling pathways and ectopic cellular processes, such as the cell cycle, apoptosis, and angiogenesis.
- LncRNAs play either pro-proliferative or anti-proliferative roles in human gastrointestinal cancers.
- LncRNAs regulate proliferation through interactions with RNA targets, localization to chromatin, or binding to proteins.
- Proliferation-related lncRNAs act on key molecules to activate or inhibit specific signaling cascades.

## Open questions

- What cellular processes, e.g., the cell cycle, apoptosis, and angiogenesis, are influenced by lncRNAs to trigger malignant cell proliferation?
- Can lncRNAs from the same gene locus, e.g., 8q24, have similar functions or molecular targets?
- Can lncRNAs be used as biomarkers for gastrointestinal cancer diagnosis or prognosis? How can we use them to decrease cancer proliferation?

## Introduction

A set of transcripts termed long non-coding RNAs (lncRNAs) are some of the most commonly researched non-coding RNA networks. Because of increasingly in-depth studies, it is likely that ~70–80% of human genome can be transcribed into RNAs, and only ~2–3% of these RNAs encode proteins^1^. The vast majority of transcripts are actually non-coding RNAs^2^. The arbitrary limits of a length of 200 nucleotides and the absence of any detectable open reading frame (ORF) distinguish lncRNAs from other non-coding RNAs^3^.

Gastrointestinal cancers have high morbidity and mortality rates, which has made them a serious public health problem. They are usually diagnosed in the advanced stages of the disease and there are only limited treatment strategies available. Colorectal cancer (CRC) is the third most common cancer in the world, with ~1.4 million newly diagnosed cases in 2012. Liver cancer is the second leading cause of cancer-related deaths worldwide in men, with an estimated 745,500 deaths. Although less frequent, other gastrointestinal cancers accounted for ~723,100 deaths from gastric cancer (GC) and 400,200 deaths from esophageal cancer (EC) in 2012^4,5^.

Tumor cell proliferation is caused by dysregulated cell processes, such as cell cycle progression, apoptosis, and angiogenesis. Normal eukaryotic cells with healthy cell cycles have the ability to control the production and release of growth-promoting signals^6^. The positive control cyclin/cyclin-dependent kinase (CDK) complexes comprise different protein kinases and contribute to the initiation and progression of the cell cycle. CDK inhibitors (CDKIs), such as the ink4 (p15, p16, p18, and p19) and Cip/Kip (p21, p27, and p57) families of proteins, play opposite roles^7–9^. Ectopic regulation of either the cyclins/CDKs/CDKIs may impinge on cell proliferation and cause malignancy. LncRNAs play highly diverse roles in the regulation of many malignant cell processes, including proliferation^10,11^. This review summarizes the research progress with respect to the function of lncRNAs in the proliferation of gastrointestinal cancers, highlighting the molecular mechanisms and the recurring signaling pathways involved in these processes.

## Molecular mechanisms of lncRNAs in proliferation

**Fig. 1:**
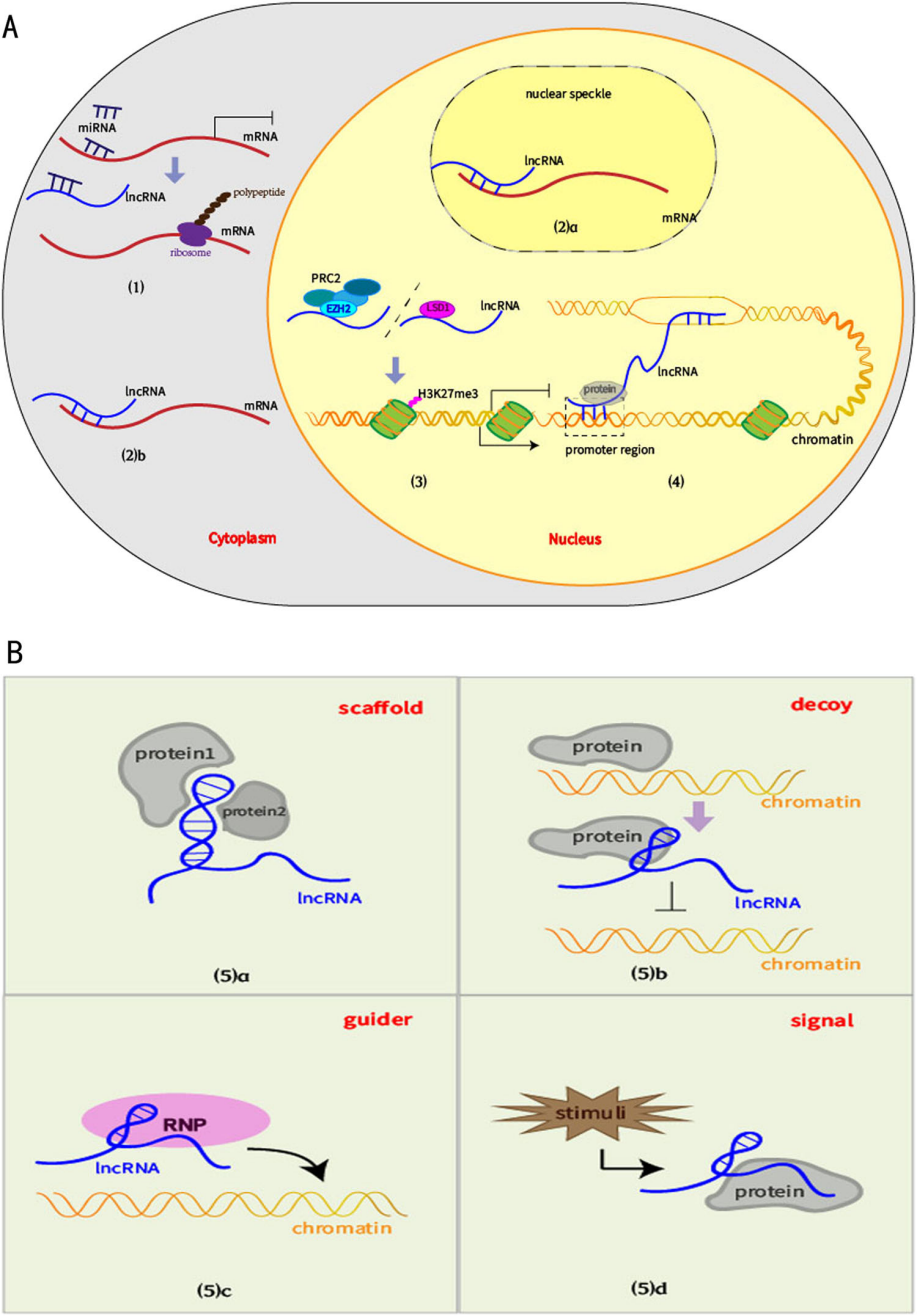
LncRNAs share common molecular mechanisms that modulate proliferation. **a**(1) LncRNAs act as ceRNAs for mRNA targets related to proliferation. (2) LncRNAs participate in mRNA metabolism involving splicing (a), mRNA stability, translation, or degradation (b). (3) LncRNAs interact with chromatin remodeling complexes such as PRC2 or LSD1. (4) LncRNAs bind to the promoter regions of neighboring genes as enhancers. **b** LncRNAs interact with proteins as (a) scaffolds to assemble multiple protein components into complexes; (b) decoys to keep proliferation-modulating proteins away from their targets; (c) guides that lead proteins such as RNPs to target sites; (d) signals with cell-type-specific expression to regulate proliferation

The function of lncRNAs in the regulation of tumor proliferation is doubled faced. On the one hand, some lncRNAs act as oncogene, promoting cell proliferation and survival, whereas, on the other hand, examples exists of lncRNAs that inhibits tumor cell growth. Consequently, lncRNAs are divided into pro-proliferative lncRNAs (Table S1) and anti-proliferative lncRNAs (Table S2). Additionally, some lncRNAs have reversed roles in different gastrointestinal cancers or adverse expression in the same cancer (Table S3). Despite their different functions, lncRNAs have common molecular mechanisms for modulating cell proliferation (Fig. 1).

The interaction between lncRNAs and other RNA targets is of great importance. The competing endogenous RNA (ceRNA)-crosstalk network has become a favorite of researchers over the past few years. CeRNAs are RNA transcripts that have special microRNA-binding sites called miRNA response elements (MREs)^12^. Many lncRNAs and mRNAs have MREs, which give them the ability to compete for shared miRNAs. In this case, lncRNAs and mRNAs can regulate each other’s expression^13–16^. In many gastrointestinal malignancies, lncRNAs may function as ceRNAs to sponge miRNAs, which liberate targeted proliferation-relevant mRNAs at the post-transcriptional level^17^. LncRNAs also engage in RNA metabolism, such as mRNA splicing, transport, translation, and degradation. When localized to nuclear speckles, where abundant spliceosomal proteins and regulatory factors accumulate, lncRNAs coordinate the alternative splicing of pre-mRNA^18^. For instance, SF2/ASF (now called SRSF1) is one of the pivotal factors co-localized with metastasis and associated with lung adenocarcinoma transcript 1 (MALAT1) in nuclear speckles. MALAT1 recruits and changes the specific distribution of SF2/ASF to promote GC proliferation^19^. Complementarily, by direct binding or specific protein induction, cytoplasmic lncRNAs participate in the modulation of either mRNA stability or translation as promoters or inhibitors^20^. In addition, the hybrid duplex between lncRNAs-AS, such as KRT7-AS, and sense mRNAs of their cognate coding genes, have recently been identified in gastrointestinal cancer growth^21^.

Some lncRNAs perform their functions when localized to chromatin^22^. Histone modification plays a central role in epigenetic transcriptional regulation. The acetylation of histones stimulates gene expression, while methylation has dual functions. Transcriptional activation is related to methylation at lysine 4, 36, and 79 of histone 3 (H3K4, H3K36, and H3K79), while silencing is associated with H3K9 and H3K27^23^. Mounting evidence indicates that numerous lncRNAs localized in the nucleus regulate cell cycle progression and proliferation by recruiting chromatin remodeling complexes and specifying histone modification patterns on target genes for their positive or negative expression. The most common remodeling complexes, e.g., methyltransferase polycomb repressive complex 2 (PRC2) and demethylase lysine-specific demethylase1 (LSD1), are regulated by lncRNAs and are responsible for silencing the expression of growth-related genes^24,25^. Nonetheless, several studies have demonstrated that upregulated zeste homolog2 (EZH2), known as a key component of PRC2, increases cyclin D1 expression and stimulates tumor cell growth, but the exact mechanism is unclear^26,27^. Gastric cancer-associated lncRNA 1 (GClnc1) is a molecular bridge that connects specific enzyme complexes, e.g., histone methyltransferase (WDR5) and acetyltransferase (KAT2A), with the target gene SOD2. SOD2 promotes proliferation because GClnc1 binds to and guides WDR5 and KAT2A to the SOD2 promoter, upregulating H3K4 trimethylation and H3K9 acetylation to improve its expression^28^.

In addition, lncRNAs actively interact with transcribed gene loci to control the expression of those genes. For instance, lncRNAs transcribed from some active chromatin states have a propensity for modulating the expression of their neighboring genes as enhancer-associated RNAs. By binding directly to the promoter sequences, these enhancer lncRNAs affect the transcription of proximal genes that are correlated tightly with cell proliferation and tumor growth in cis^22^.

Many studies have underscored the significance of crosstalk with diverse proteins. When lncRNAs interact with proteins, they function as scaffolds, guides, decoys, and signals^29^. LncRNA scaffolds with complicated and functional three-dimensional structures serve as a platform to collect regulatory protein partners, such as LSD1 and PRC2, to promoter regions of proliferation-related genes. Moreover, the formation of the complex including lncRNA and ribonucleoprotein (RNP), e.g., hnRNP-K, suggests that lncRNAs serve as guides that lead proteins to their target sites^30^. Some lncRNAs, such as the UHRF1 protein-associated transcript (UPAT), influence growth by modulating protein ubiquitination and degradation^31^. LncRNAs inhibit pro-ubiquitination proteins by acting as decoys that prevent them from executing their functions. Additionally, when confronted with various stimuli, some lncRNAs function as molecular signals, exhibiting cell-type-specific expression and regulating biological events involving the cell cycle or apoptosis^29^.

## LncRNAs govern proliferation through different signaling pathways

The malignant proliferation of gastrointestinal tumor cells is always accompanied by the dysregulation of multiple signaling pathways. LncRNAs mediate cell growth by acting on key molecules to activate or restrain specific signaling pathways.

### P53 signaling pathway

**Fig. 2:**
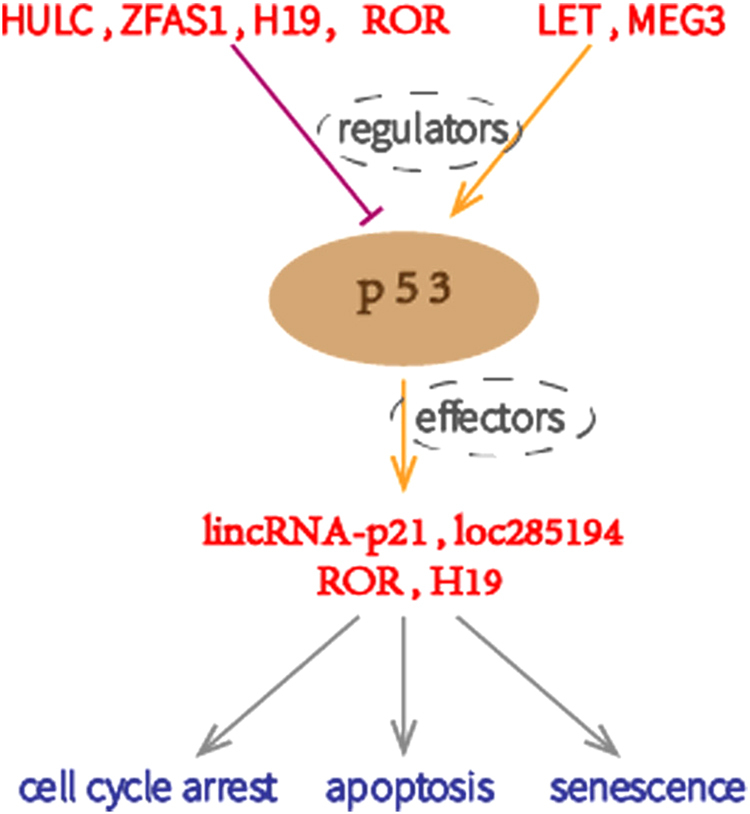
Proliferation-associated lncRNAs act as p53 regulators or effectors by activating or inhibiting the p53 signaling pathway

As a canonical suppressor and crucial cell cycle gatekeeper, p53 is capable of suspending the cell cycle when eukaryotic cells suffer from drastic DNA damage or deficient oxygenation. Nonetheless, if the stress signals appear overwhelming and irreparable, p53 tends to induce permanent cell cycle arrest (cellular senescence) or apoptosis instead^32^. The p53 transcriptional response during carcinogenesis and tumor cell proliferation includes the activation and inhibition of various genes. The repressive function of p53 is closely correlated to that of lncRNAs^33^. LncRNAs regulate p53 and are key components of the p53 pathway (Fig. 2)^34^.

p53 effectors are those lncRNAs coordinated by p53. There is a reciprocal repression feedback loop between loc285194 (LSAMP antisense RNA 3 or TUSC7) and microRNAs that belong to the ceRNA crosstalk networks, and loc285194 has a consensus response element (p53RE) in its promoter that binds directly to p53. In this way, loc285194 functions as a downstream p53 effector that exerts its anti-proliferative role by binding miR-211 in CRC and miR-23b in GC^35,36^. Another representative lncRNA is intergenic lincRNA-p21.

When confronted with DNA damage, the activated p53 signal induces lincRNA-p21, which executes suppressive functions at both the transcriptional and post-transcriptional levels^37^. In the nucleus, lincRNA-p21 promotes its neighboring gene p21 as an RNA enhancer and downregulates various p53 target genes to facilitate apoptosis by directly binding to hnRNP-K^38^. In the cytoplasm, however, lincRNA-p21 degrades in an HuR-dependent manner to de-repress Rck, which activates β-catenin and the cell cycle catalyst JunB^39,40^.

Other lncRNAs also regulate p53. The tumor suppressor role of maternally expressed gene 3 (MEG3) and long non-coding RNA-low expression in tumor (lncRNA-LET) is evident in several gastrointestinal cancers. Data from other studies indicate that the anti-proliferative function of lncRNAs is partially accomplished by activating the p53 pathway^41–46^. In CRC and hepatocellular carcinoma (HCC), zinc finger antisense 1 (ZFAS1) destabilizes p53 to modulate CDK1 and the expression of its partner, cyclin B1, thus facilitating the G1/S transition, while highly upregulated in liver cancer (HULC) represses its neighboring eukaryotic translation elongation factor 1 epsilon 1 (EEF1E), which is a pivotal p53 activator and this mechanism promotestumor cell growth^47,48^.

LncRNA-ROR and lncRNA-H19 can be both regulators and effectors of p53 in gastrointestinal cancer proliferation. Overexpressed ROR increases CRC growth by inhibiting p53. Studies have shown that lncRNA-ROR is under the control of p53 in response to DNA damage^49,50^. LncRNA-H19 is only activated by p53 through the HIF pathway in HCC but, in GC, it precludes p53 activity and represses Bax expression to avoid apoptosis^51^.

### Akt signaling pathway

**Fig. 3:**
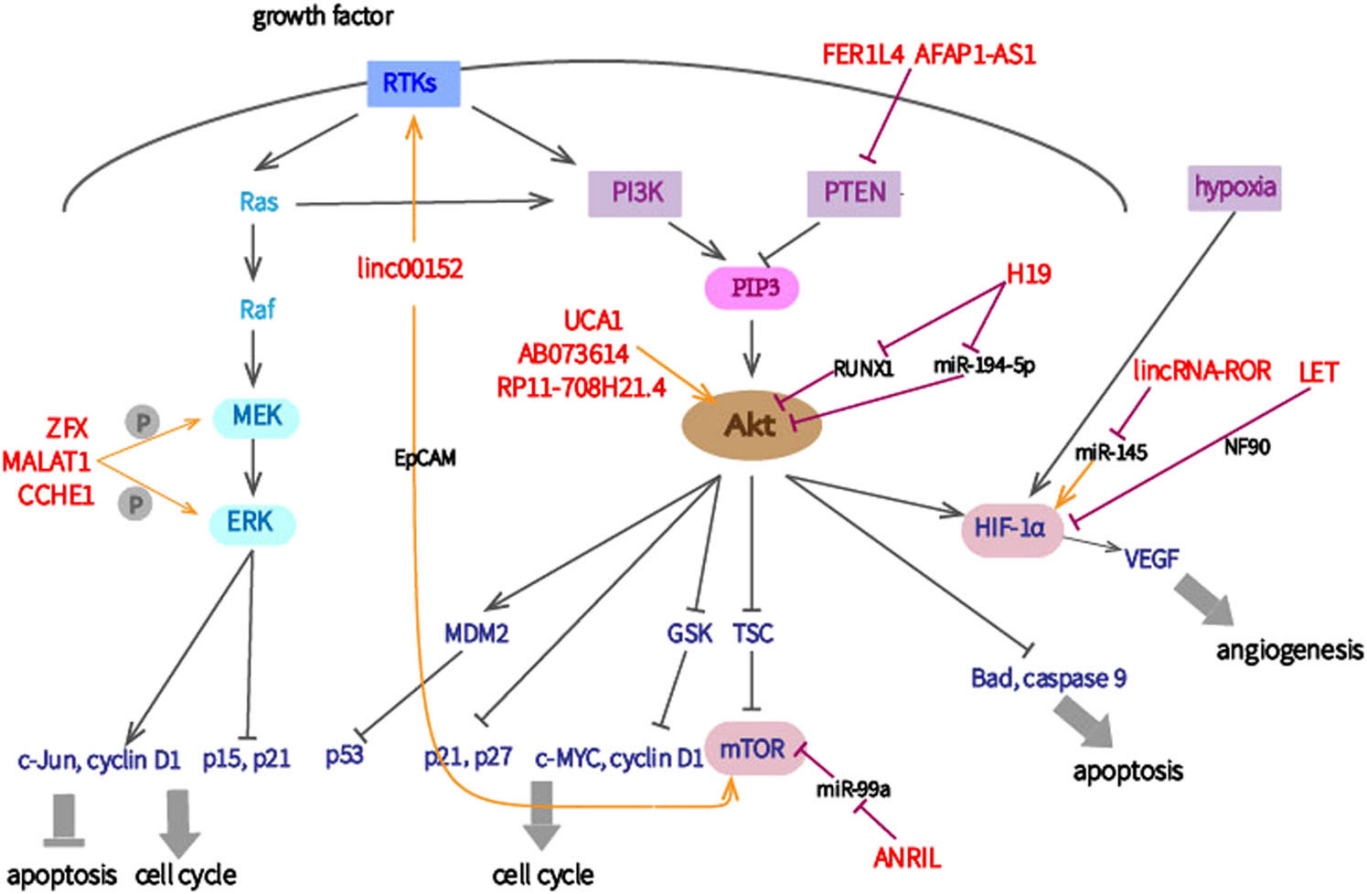
Proliferation-associated lncRNAs act at specific functional sites of the Akt and MAPK signaling pathways

Akt signaling role in tumorigenesis is well established^52^. Activated by growth factors or angiogenesis inducers through receptor tyrosine kinases (RTKs), PI3K phosphorylates PIP2 to produce PIP3, while the phosphatase and tensin homolog deleted on chromosome 10 (PTEN) inhibits this process. Like RTKs, PI3K is also a direct effector of the Ras protein, which provides crosstalk with the MAPK signaling pathway^52,53^. Additionally, protein kinase B (PKB/Akt) and mammalian target of rapamycin (mTOR) play crucial roles in cell growth stimulation^12^. By inhibiting the expression of cyclin D1, Bcl-2 antagonist of cell death (Bad), and caspase-9 by increasing their phosphorylation levels, Akt promotes the G1/S transition and prevents tumor cells from undergoing apoptosis. The concomitant activation of HIF-1 and vascular endothelial growth factor (VEGF) corroborate the involvement of Akt in angiogenesis to indirectly facilitate proliferation (Fig. 3)^54,55^.

Ectopic expression of linc00152 accelerates cell proliferation in many gastrointestinal cancers^56^. Linc00152 directly binds and activates the epithelial growth factor receptor (EGFR), which is a member of the RTK family^52,57^. In HCC, linc00152 activates the PI3K/Akt/mTOR cascades by binding directly to its neighbor, then TOR-related gene EpCAM through cis-regulation^58^. In gallbladder cancer (GBC), overexpressed linc00152, induced by transcription factor specificity protein 1(SP1), promotes proliferation via the PI3K/Akt pathway^56^. Another lncRNA, urothelial carcinoma-associated 1 (UCA1), is highly expressed in GC. UCA1 elevates cyclin D1 expression and the G1/S transition by binding directly to EZH2 and promoting interaction with cyclin D1 promoter and also by activating the AKT/GSK3β/cyclin D1 axis. Furthermore, EZH2 and phosphorylated Akt can influence the expression of each other to form a positive feedback loop. In conclusion, UCA1 stimulates proliferation by increasing EZH2 and activating the Akt pathway^26,27,59^. In terms of lncRNA-H19, the expression of the tumor suppressor RUNX1 is downregulated by the lncRNA-H19/miR-675 axis to induce Akt/mTOR signal activation; however, in GBC, lncRNA-H19 acts as a ceRNA for miR-194-5p to liberate Akt2^60–62^.

Like PI3K, PTEN is a well-known regulator of Akt. The pseudogene-derived lncRNA fer-1-like family member 4 (lncRNA-FER1 L4) is strongly downregulated in GC and induces cell cycle arrest. Because FER1 L4 and PTEN mRNA both target miR-106a-5p and because PTEN is a cell cycle inhibitor, it is likely that the anti-proliferative role of FER1 L4 occurs partially through the PTEN/Akt pathway^63^. Upregulated AFAP1-AS1 may mediate GC proliferation and apoptosis by the same mechanism^64^. The lncRNAs AB073614 and RP11-708H21.4 are highly expressed in CRC and stimulate proliferation and hamper apoptosis through the Akt signaling pathway, while the exact lncRNA functional molecules warrant further investigation^65,66^.

### MAPK signaling pathway

Mitogen-activated protein kinases (MAPK) are part of the serine–threonine kinase family and they participate extensively in tumorigenesis. Activation of the MAPK pathway, by phosphorylating MAPK kinases, MEK1/2, and extracellular-signal-regulated kinases (ERK1/2), contributes to perpetual cell growth and survival^53^. ERKs coordinate the synthesis of cyclin D1 and the degradation of the INK4 proteins, such as CDKN1A (p21) and CDKN2B (p15) (Fig. 3)^67^.

MALAT1, which is localized on chromosome 11q13.1, is highly expressed in gastrointestinal cancers. In GBC, the knockdown of MALAT1 significantly impairs the expression of phosphorylated MEK1/2, ERK1/2, and JNK without any changes in quantity, suggesting that this lncRNA is likely to improve GBC proliferation by activating the ERK/MAPK signaling pathway^68^. Upregulated zinc finger protein X-linked (ZFX) and cervical carcinoma expressed PCNA regulatory lncRNA (CCHE1) increase cell cycle progression and decrease apoptosis to stimulate proliferation because of their similar mechanisms in GC and HCC, respectively^69,70^. Nevertheless, the direct link between lncRNAs and the MAPK pathway remains enigmatic.

### Wnt/β-catenin signaling pathway

**Fig. 4:**
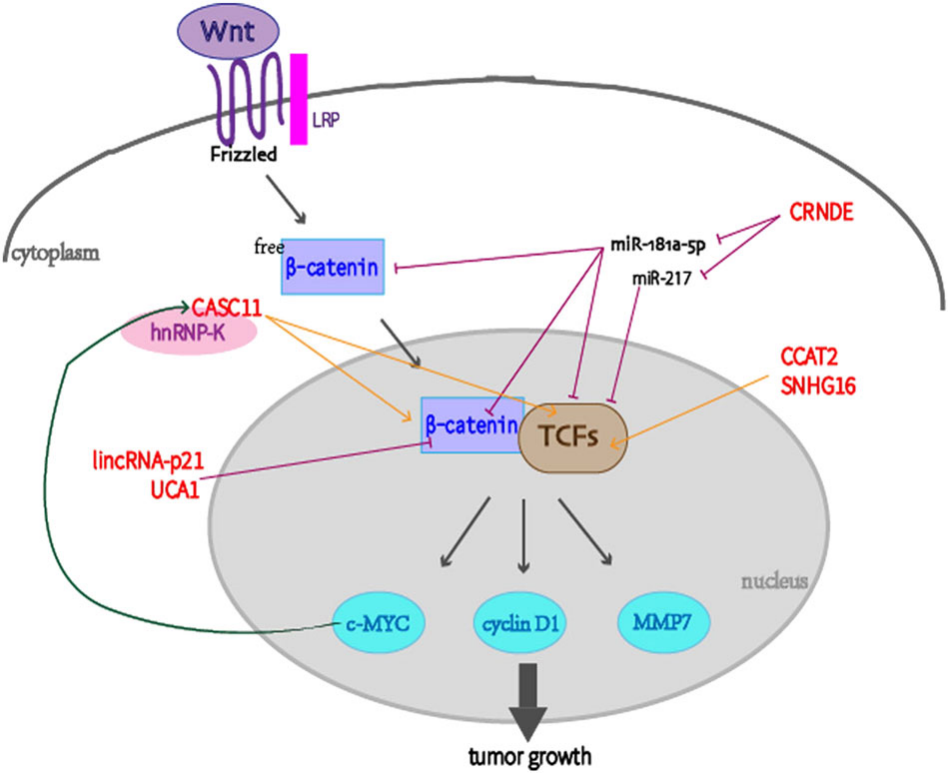
Proliferation-associated lncRNAs act on at specific functional sites of the Wnt signaling pathway

The Wnt signaling pathway can be categorized as either canonical or non-canonical based on its downstream components. Here, we focus on the former because of its important role in gastrointestinal cancer initiation and progression^71^. The activation of the canonical Wnt pathway, accurately termed the Wnt/β-catenin/TCF signaling pathway, mainly depends on translocation of the central oncogene, β-catenin. Both negative mutation of adenomatous polyposis coli (APC) oraxin and positive mutation of β-catenin itself can lead to cytoplasmic accumulation of free β-catenin and its translocation to the nucleus to bind to T cell factors (TCFs). Consequently, a series of downstream targets, including c-MYC, cyclin D1, MMP7, and ITF-2, undergo transcriptional activation^72^. LncRNAs interact with critical components of the canonical Wnt pathway and are therefore involved in the regulation of cell proliferation (Fig. 4).

LncRNA colorectal neoplasia differentially expressed (CRNDE) is upregulated in CRC and can act as ceRNA for different miRNAs to promote tumor proliferation. Han et al. demonstrated that β-catenin and TCF4 are targets of miR-181a-5p, which inhibits their expression. Similarly, miR-217 modulates transcription factor 7-like 2 (TCF7 L2)^73,74^. Because overexpressed CRNDE hampers specific miRNAs that inhibits β-catenin, TCFs, c-MYC, and cyclin D1, its pro-proliferation function is partially exerted through the Wnt/β-catenin pathway.

In addition to inducing miRNA expression, proteins can also serve as bridges between lncRNAs and key components of the Wnt pathway. HnRNP-K, a protein with a nuclear shutting domain (KAS), interacts with β-catenin and TCF4 to facilitate the import and nuclear accumulation of β-catenin. A positive feedback loop between CASC11 and hnRNP-K provides an alternative explanation for how the upregulation of CASC11 activates the Wnt/β-catenin pathway to stimulate CRC growth through the induction of hnRNP-K. c-MYC, an important target of β-catenin, increases CASC11 expression through epigenetic regulation^75^.

LncRNAs stimulate the Wnt signaling pathway through direct physical interactions with key effectors. Ling et al. confirmed that CCAT2 increases TCF7 L2 transcriptional activity without affecting its quantity in CRC. Furthermore, CCAT2 is likely to be a downstream target of the Wnt pathway because of the TCF7 L2-binding element within its SNP loci. This positive feedback loop supports the function of highly expressed CCAT2 in CRC growth^76^. Similarly, the combination of lincRNA-p21 and its mRNA target, CTNNB1, precludes the translation of β-catenin, which suggests that the anti-proliferative role of lincRNA-p21 in CRC is accomplished through the canonical Wnt pathway^39^. The snoRNA host gene 16 (SNHG16) is regulated by Wnt targets, including TCF1, TCF4, and c-MYC, and its knockdown promotes CRC cell death and apoptosis. This presents the possibility that upregulated SNHG16 may stimulate tumor proliferation via the Wnt pathway^77^. Data from Wang et al. demonstrate that the expression of lncRNA UCA1 is reduced in esophageal squamous cell carcinoma (ESCC) and overexpressed UCA1 decreases the levels of the active form of nuclear β-catenin. So, UCA1 may inhibit ESCC growth through the Wnt/ β-catenin signaling pathway^78^.

### MYC signaling pathway

**Fig. 5:**
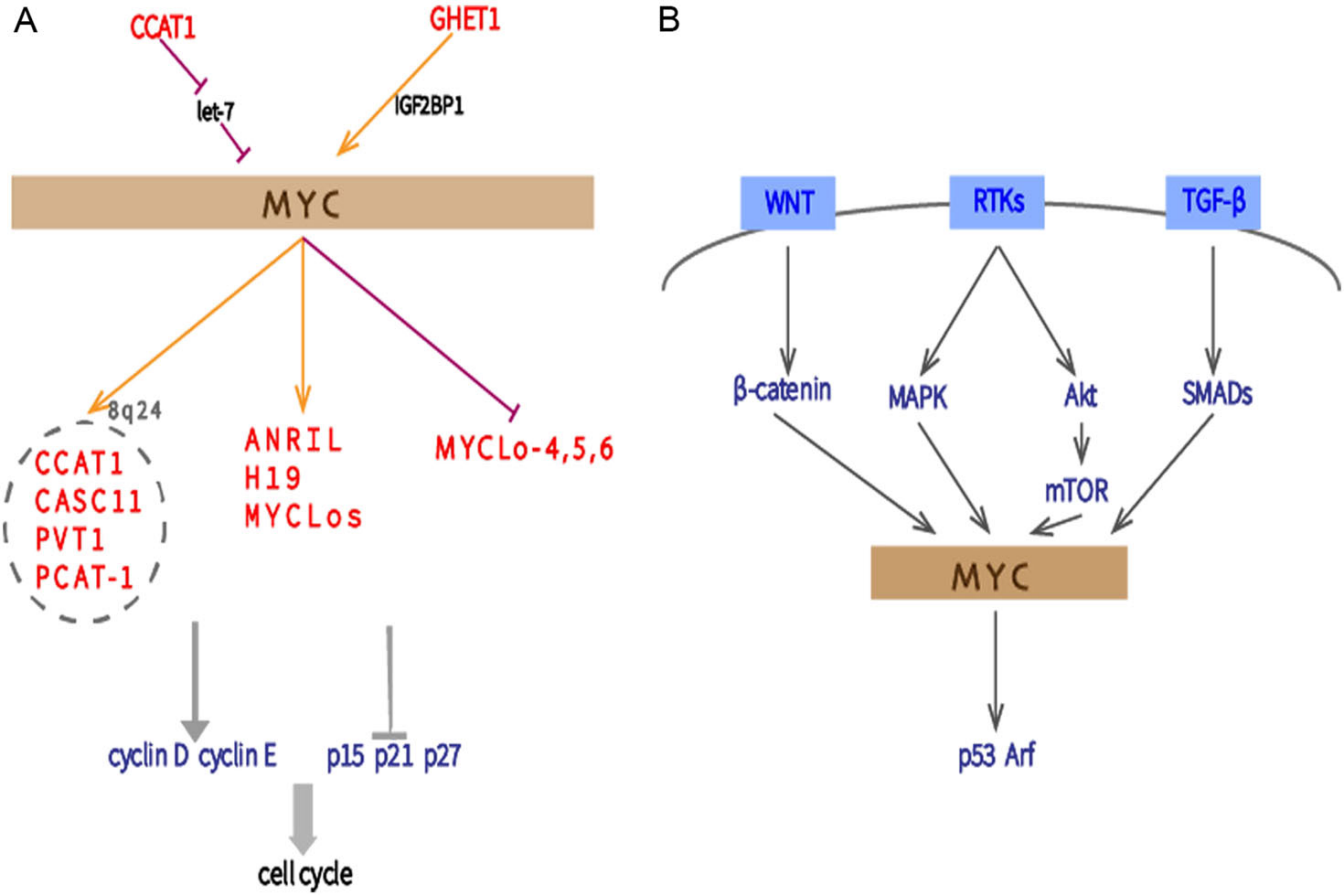
**a** Proliferation-associated lncRNAs act as MYC regulators or effectors by activating or inhibiting the MYC signaling pathway. **b** MYC lies at the crossroads of the Wnt, Akt, MAPK, TGF-β, and p53 signaling pathways

The proto-oncogene MYC is frequently upregulated in gastrointestinal cancers, and its gene product is a transcription factor that can accelerate cell proliferation by regulating the expression of genes involved in cell cycle progression^79^. It upregulates the expression of cyclin D and cyclin E, which both promote cell cycle progression. Myc also hampers expression of crucial cyclin/CDK inhibitors, such as CDKN2B (p15), CDKN1A (p21), and CDKN1B (p27)^80^. MYC lies at the crossroads of the pathways involved in regulating cellular proliferation. It is a downstream target of the Wnt, Akt, MAPK, and TGF-β signaling cascades and activates the p53 and ARF checkpoints, which makes it even more important to cell growth regulation (Fig. 5b)^81^. LncRNAs both support and repress the pro-proliferative function of MYC by regulating the MYC-target cell cycle modulator genes (Fig. 5a).

Colon cancer-associated transcript 1 (CCAT1), CASC11, plasmacytoma variant translocation 1 (PVT1), and PCAT-1 are lncRNAs that are all localized on the gene desert of chromosome 8q24 and interact with MYC. Among them, CCAT1 and CASC11 can be induced by MYC due to the presence of an E-box Myc-responsive element in their promoters^75,82–84^. In CRC, the PVT1 expression correlates with c-MYC expression levels. Similarly, MYC-related lncRNAs MYCLos are overexpressed in CRC and sustain MYC-driven tumorigenesis. Indeed, MYCLos-1 represses p21, whereas MYCLos-2 (CCAT6) represses p15 by interacting with the HuR and hnRNP-K proteins, respectively^79^. By binding to the promoter of LncRNA-H19 in GC or of HOTAIR in GBC, c-MYC increases their expression^43,85^. In some instances, MYC can also restrict lncRNA expression. For example, lncRNAs MYCLo-4, -5, and -6, with known tumor-suppressive roles in CRC, are repressed by MYC^86^.

Notably, MYC can be characterized as a downstream effector of lncRNA. For instance, lncRNA gastric carcinoma high expressed transcript 1 (GHET1) was found to be upregulated in GC. By binding to insulin-like growth factor 2 mRNA-binding protein 1 (IGF2BP1), GHET1 increases the physical interaction between c-Myc mRNA and IGF2BP1, therefore improves the stability of c-Myc mRNA and upregulates its expression. The expression of GHET1 and c-Myc are strongly associated and link with malignant progression in GC^87^.

### Other signaling pathways

The distribution of regulatory T cells (T-regs) is highly necessary in immune processes and tumorigenesis. T-reg differentiation, TGF-β recruitment, and the subsequent increase in phosphorylated SMAD3 and SMAD2 induced by linc-POU3F3, result in a pro-proliferative role in GC^88^. Takahashi et al. reported that knockdown of PVT1 in CRC could activate several genes of the TGF-β signaling pathway, including SMAD4 and ROCK1. These genes are capable of inducing apoptosis and invasion^89^. Moreover, a feedback loop between TGF-β1 and PVT1 in HCC has been suggested, as PVT1 can not only be induced by TGF-β1 but, in turn, it also activates the TGF-β signaling pathway^90^.

The hypoxia-inducible factor-1 (HIF-1) sustains cell proliferation and survival, and angiogenesis in hypoxic tumor areas. A crosstalk between the HIF-1 pathway and lncRNA network exists^6,54^. LncRNA-ROR sponges miR-145 to increase HIF-1α expression and therefore expression of its downstream target VEGF^49^. lncRNA-LET stabilizes NF90 (a double-stranded RNA-binding protein) to improve HIF-1α mRNA stability in GBC, while p53 increases H19 expression through the HIF signaling pathway in HCC^43,45,51^.

The previously discussed lncRNA MALAT1, which is overexpressed in ESCC, induces dephosphorylation of the ATM/CHK2 pathway thereby overcoming the G2/M cell cycle checkpoint^91^. In addition, MEG3 and lincRNA-p21 contribute to apoptosis and growth inhibition through endoplasmic reticulum (ER) stress pathways in ESCC and HCC, respectively^39,92^. In GC, lncRNA gastric cancer-associated transcript 3 (GACAT3) works as a downstream target of IL6/STAT3 signaling pathway to accomplish its function^93^.

## Conclusions

Although advancements in surveying the molecular mechanisms underlying the actions of proliferation-associated lncRNAs have broadened our ability to annotate these tumor-related transcripts, the exact signaling pathways in which many lncRNAs participate remain unclearly defined. The major signaling cascades and specific functional sites reviewed here may provide a foundation upon which new lncRNAs involved in gastrointestinal tumor proliferation can be identified and may motivate an increased focus on understanding the signaling pathways, which remain enigmatic. Malignant cell growth can be non-comprehensive because it can be caused by any of several dysregulated cellular processes, such as the cell cycle, apoptosis, angiogenesis, and cell senescence; it is likely that the lncRNAs described here are important regulators of these pathophysiological processes. Consequently, lncRNAs characterized as oncogenes or tumor suppressors that modulate proliferation may be promising biomarkers and therapeutic targets in gastrointestinal cancers^41,94–170^.

## Electronic supplementary material

Table S1

Table S2

Table S3

## References

[1] Kung JT, Colognori D, Lee JT (2013). Long noncoding RNAs: past, present, and future. Genetics.

[2] Eddy SR (2001). Non-coding RNA genes and the modern RNA world. Nat. Rev. Genet..

[3] Tano K, Akimitsu N (2012). Long non-coding RNAs in cancer progression. Front. Genet..

[4] Chen W (2016). Cancer statistics in China, 2015. CA Cancer J. Clin..

[5] Torre LA (2015). Global cancer statistics, 2012. CA Cancer J. Clin..

[6] Hanahan D, Weinberg RA (2011). Hallmarks of cancer: the next generation. Cell.

[7] Noatynska A, Tavernier N, Gotta M, Pintard L (2013). Coordinating cell polarity and cell cycle progression: what can we learn from flies and worms?. Open Biol..

[8] Soufi A, Dalton S (2016). Cycling through developmental decisions: how cell cycle dynamics control pluripotency, differentiation and reprogramming. Development.

[9] Kapinas K (2013). The abbreviated pluripotent cell cycle. J. Cell. Physiol..

[10] Nagano T, Fraser P (2011). No-nonsense functions for long noncoding RNAs. Cell.

[11] Lee JT (2012). Epigenetic regulation by long noncoding RNAs. Science.

[12] Tay Y, Rinn J, Pandolfi PP (2014). The multilayered complexity of ceRNA crosstalk and competition. Nature.

[13] Salmena L, Poliseno L, Tay Y, Kats L, Pandolfi PP (2011). A ceRNA hypothesis: the Rosetta Stone of a hidden RNA language?. Cell.

[14] Guo LL (2015). Competing endogenous RNA networks and gastric cancer. World J. Gastroenterol..

[15] Paraskevopoulou MD (2013). DIANA-LncBase: experimentally verified and computationally predicted microRNA targets on long non-coding RNAs. Nucleic Acids Res..

[16] Friedman RC, Farh KK, Burge CB, Bartel DP (2009). Most mammalian mRNAs are conserved targets of microRNAs. Genome Res..

[17] Filipowicz W, Bhattacharyya SN, Sonenberg N (2008). Mechanisms of post-transcriptional regulation by microRNAs: are the answers in sight?. Nat. Rev. Genet..

[18] Yoshimoto R, Mayeda A, Yoshida M, Nakagawa S (2016). MALAT1 long non-coding RNA in cancer. Biochim. Biophys. Acta.

[19] Wang J (2014). MALAT1 promotes cell proliferation in gastric cancer by recruiting SF2/ASF. Biomed. Pharmacother..

[20] Batista PJ, Chang HY (2013). Long noncoding RNAs: cellular address codes in development and disease. Cell.

[21] Huang B (2016). Long non-coding antisense RNA KRT7-AS is activated in gastric cancers and supports cancer cell progression by increasing KRT7 expression. Oncogene.

[22] Schmitt AM, Chang HY (2016). Long noncoding RNAs in cancer pathways. Cancer Cell.

[23] Kouzarides T (2007). Chromatin modifications and their function. Cell.

[24] Simon JA, Kingston RE (2009). Mechanisms of polycomb gene silencing: knowns and unknowns. Nat. Rev. Mol. Cell Biol..

[25] Jin Y (2017). LSD1 collaborates with EZH2 to regulate expression of interferon-stimulated genes. Biomed. Pharmacother..

[26] Cao W (2012). Up-regulation of enhancer of zeste homolog 2 is associated positively with cyclin D1 overexpression and poor clinical outcome in head and neck squamous cell carcinoma. Cancer.

[27] Wang ZQ (2017). Long noncoding RNA UCA1 induced by SP1 promotes cell proliferation via recruiting EZH2 and activating AKT pathway in gastric cancer. Cell Death Dis..

[28] Sun TT (2016). LncRNA GClnc1 promotes gastric carcinogenesis and may act as a modular scaffold of WDR5 and KAT2A complexes to specify the histone modification pattern. Cancer Discov..

[29] Wang KC, Chang HY (2011). Molecular mechanisms of long noncoding RNAs. Mol. Cell.

[30] Rinn JL, Chang HY (2012). Genome regulation by long noncoding RNAs. Annu. Rev. Biochem..

[31] Taniue K (2016). Long noncoding RNA UPAT promotes colon tumorigenesis by inhibiting degradation of UHRF1. Proc. Natl Acad. Sci. USA.

[32] Bieging KT, Mello SS, Attardi LD (2014). Unravelling mechanisms of p53-mediated tumour suppression. Nat. Rev. Cancer.

[33] Huarte M (2010). A large intergenic noncoding RNA induced by p53 mediates global gene repression in the p53 response. Cell.

[34] Zhang A, Xu M, Mo YY (2014). Role of the lncRNA-p53 regulatory network in cancer. J. Mol. Cell Biol..

[35] Liu Q (2013). LncRNA loc285194 is a p53-regulated tumor suppressor. Nucleic Acids Res..

[36] Qi P (2015). Reciprocal repression between TUSC7 and miR-23b in gastric cancer. Int. J. Cancer.

[37] Chaudhary, R. & Lal, A. Long noncoding RNAs in the p53 network. *Wiley Interdiscip. Rev. RNA* **8**, e1410 (2017). 10.1002/wrna.1410 PMC6314037 27990773

[38] Grossi E, Sanchez Y, Huarte M (2016). Expanding the p53 regulatory network: LncRNAs take up the challenge. Biochim. Biophys. Acta.

[39] Chen S (2017). LincRNa-p21: function and mechanism in cancer. Med. Oncol..

[40] Yoon JH (2012). LincRNA-p21 suppresses target mRNA translation. Mol. Cell.

[41] Zhuo H (2016). The aberrant expression of MEG3 regulated by UHRF1 predicts the prognosis of hepatocellular carcinoma. Mol. Carcinog..

[42] Braconi C (2011). microRNA-29 can regulate expression of the long non-coding RNA gene MEG3 in hepatocellular cancer. Oncogene.

[43] Khandelwal A, Malhotra A, Jain M, Vasquez KM, Jain A (2017). The emerging role of long non-coding RNA in gallbladder cancer pathogenesis. Biochimie.

[44] Wang PL (2016). Long non-coding RNA-low expression in tumor inhibits the invasion and metastasis of esophageal squamous cell carcinoma by regulating p53 expression. Mol. Med. Rep..

[45] Yang F (2013). Repression of the long noncoding RNA-LET by histone deacetylase 3 contributes to hypoxia-mediated metastasis. Mol. Cell.

[46] Tian J (2017). Identification of the long noncoding RNA LET as a novel tumor suppressor in gastric cancer. Mol. Med. Rep..

[47] Thorenoor N (2016). Long non-coding RNA ZFAS1 interacts with CDK1 and is involved in p53-dependent cell cycle control and apoptosis in colorectal cancer. Oncotarget.

[48] Yu X, Zheng H, Chan MT, Wu WK (2017). HULC: an oncogenic long non-coding RNA in human cancer. J. Cell. Mol. Med..

[49] Pan Y (2016). The emerging roles of long noncoding RNA ROR (lincRNA-ROR) and its possible mechanisms in human cancers. Cell. Physiol. Biochem..

[50] Li H, Jiang X, Niu X (2017). Long non-coding RNA reprogramming (ROR) promotes cell proliferation in colorectal cancer via affecting P53. Med. Sci. Monit..

[51] Zhang L (2017). The interplay of LncRNA-H19 and its binding partners in physiological process and gastric carcinogenesis. Int. J. Mol. Sci..

[52] Khan KH, Yap TA, Yan L, Cunningham D (2013). Targeting the PI3K-AKT-mTOR signaling network in cancer. Chin. J. Cancer.

[53] De Luca A, Maiello MR, D’Alessio A, Pergameno M, Normanno N (2012). The RAS/RAF/MEK/ERK and the PI3K/AKT signalling pathways: role in cancer pathogenesis and implications for therapeutic approaches. Expert Opin. Ther. Targets.

[54] Sasaki T, Kuniyasu H (2014). Significance of AKT in gastric cancer (Review). Int. J. Oncol..

[55] Tokunaga E (2008). Deregulation of the Akt pathway in human cancer. Curr. Cancer Drug Targets.

[56] Yu, Y. et al. LINC00152: a pivotal oncogenic long non-coding RNA in human cancers. *Cell Prolif*. **50**, e12349 (2017). 10.1111/cpr.12349 PMC6529135 28464433

[57] Zhou J (2015). Linc00152 promotes proliferation in gastric cancer through the EGFR-dependent pathway. J. Exp. Clin. Cancer Res..

[58] Ji J (2015). LINC00152 promotes proliferation in hepatocellular carcinoma by targeting EpCAM via the mTOR signaling pathway. Oncotarget.

[59] Geng J (2015). EZH2 promotes tumor progression via regulating VEGF-A/AKT signaling in non-small cell lung cancer. Cancer Lett..

[60] Liu G, Xiang T, Wu QF, Wang WX (2016). Long noncoding RNA H19-derived miR-675 enhances proliferation and invasion via RUNX1 in gastric cancer cells. Oncol. Res..

[61] Zhuang M, Gao W, Xu J, Wang P, Shu Y (2014). The long non-coding RNA H19-derived miR-675 modulates human gastric cancer cell proliferation by targeting tumor suppressor RUNX1. Biochem. Biophys. Res. Commun..

[62] Wang SH (2016). Long noncoding RNA H19 contributes to gallbladder cancer cell proliferation by modulated miR-194-5p targeting AKT2. Tumour Biol..

[63] Xia T (2015). Long noncoding RNA FER1L4 suppresses cancer cell growth by acting as a competing endogenous RNA and regulating PTEN expression. Sci. Rep..

[64] Guo JQ, Li SJ, Guo GX (2017). Long noncoding RNA AFAP1-AS1 promotes cell proliferation and apoptosis of gastric cancer cells via PTEN/p-AKT pathway. Dig. Dis. Sci..

[65] Wang Y (2017). LncRNA AB073614 regulates proliferation and metastasis of colorectal cancer cells via the PI3K/AKT signaling pathway. Biomed. Pharmacother..

[66] Sun L (2017). Down-regulation of long non-coding RNA RP11-708H21.4 is associated with poor prognosis for colorectal cancer and promotes tumorigenesis through regulating AKT/mTOR pathway. Oncotarget.

[67] Fang JY, Richardson BC (2005). The MAPK signalling pathways and colorectal cancer. Lancet Oncol..

[68] Wu XS (2014). MALAT1 promotes the proliferation and metastasis of gallbladder cancer cells by activating the ERK/MAPK pathway. Cancer Biol. Ther..

[69] Peng W, Fan H (2016). Long noncoding RNA CCHE1 indicates a poor prognosis of hepatocellular carcinoma and promotes carcinogenesis via activation of the ERK/MAPK pathway. Biomed. Pharmacother..

[70] Wu S (2013). Knockdown of ZFX inhibits gastric cancer cell growth in vitro and in vivo via downregulating the ERK-MAPK pathway. Cancer Lett..

[71] Doucas H, Garcea G, Neal CP, Manson MM, Berry DP (2005). Changes in the Wnt signalling pathway in gastrointestinal cancers and their prognostic significance. Eur. J. Cancer.

[72] Kolligs FT, Bommer G, Goke B (2002). Wnt/beta-catenin/tcf signaling: a critical pathway in gastrointestinal tumorigenesis. Digestion.

[73] Yu B (2017). The long non-coding RNA CRNDE promotes colorectal carcinoma progression by competitively binding miR-217 with TCF7L2 and enhancing the Wnt/beta-catenin signaling pathway. Cell. Physiol. Biochem..

[74] Han P (2017). The lncRNA CRNDE promotes colorectal cancer cell proliferation and chemoresistance via miR-181a-5p-mediated regulation of Wnt/beta-catenin signaling. Mol. Cancer.

[75] Zhang Z (2016). Long non-coding RNA CASC11 interacts with hnRNP-K and activates the WNT/β-catenin pathway to promote growth and metastasis in colorectal cancer. Cancer Lett..

[76] Ling H (2013). CCAT2, a novel noncoding RNA mapping to 8q24, underlies metastatic progression and chromosomal instability in colon cancer. Genome Res..

[77] Christensen LL (2016). SNHG16 is regulated by the Wnt pathway in colorectal cancer and affects genes involved in lipid metabolism. Mol. Oncol..

[78] Wang X (2016). lncRNA UCA1 inhibits esophageal squamous-cell carcinoma growth by regulating the Wnt signaling pathway. J. Toxicol. Environ. Health A.

[79] Kim, T. et al. Role of MYC-regulated long noncoding RNAs in cell cycle regulation and tumorigenesis. *J. Natl Cancer Inst*. **107**, dju505 (2015). 10.1093/jnci/dju505 PMC4402359 25663692

[80] Gandarillas A (2012). The mysterious human epidermal cell cycle, or an oncogene-induced differentiation checkpoint. Cell Cycle.

[81] Wan X (2015). The functional sites of miRNAs and lncRNAs in gastric carcinogenesis. Tumour Biol..

[82] Zhou, D. D., Liu, X. F., Lu, C. W., Pant, O. P. & Liu, X. D. Long non-coding RNA PVT1: emerging biomarker in digestive system cancer. *Cell Prolif*. **50**, e12398 (2017). 10.1111/cpr.12398 PMC6529066 29027279

[83] Qiao L (2017). Down regulation of the long non-coding RNA PCAT-1 induced growth arrest and apoptosis of colorectal cancer cells. Life Sci..

[84] Xin Y, Li Z, Shen J, Chan MT, Wu WK (2016). CCAT1: a pivotal oncogenic long non-coding RNA in human cancers. Cell Prolif..

[85] Zhang EB (2014). c-Myc-induced, long, noncoding H19 affects cell proliferation and predicts a poor prognosis in patients with gastric cancer. Med. Oncol..

[86] Kim T (2015). MYC-repressed long noncoding RNAs antagonize MYC-induced cell proliferation and cell cycle progression. Oncotarget.

[87] Yang F (2014). Long non-coding RNA GHET1 promotes gastric carcinoma cell proliferation by increasing c-Myc mRNA stability. FEBS J..

[88] Xiong G, Yang L, Chen Y, Fan Z (2015). Linc-POU3F3 promotes cell proliferation in gastric cancer via increasing T-reg distribution. Am. J. Transl. Res..

[89] Takahashi Y (2014). Amplification of PVT-1 is involved in poor prognosis via apoptosis inhibition in colorectal cancers. Br. J. Cancer.

[90] Wang F (2014). Oncofetal long noncoding RNA PVT1 promotes proliferation and stem cell-like property of hepatocellular carcinoma cells by stabilizing NOP2. Hepatology.

[91] Hu L (2015). Up-regulation of long noncoding RNA MALAT1 contributes to proliferation and metastasis in esophageal squamous cell carcinoma. J. Exp. Clin. Cancer Res..

[92] Huang ZL (2017). Long non-coding RNA MEG3 induces cell apoptosis in esophageal cancer through endoplasmic reticulum stress. Oncol. Rep..

[93] Shen W (2016). Novel long non-coding RNA GACAT3 promotes gastric cancer cell proliferation through the IL-6/STAT3 signaling pathway. Tumour Biol..

[94] Guo K (2017). The expression pattern of long non-coding RNA PVT1 in tumor tissues and in extracellular vesicles of colorectal cancer correlates with cancer progression. Tumour Biol..

[95] Jin J, Chu Z, Ma P, Meng Y, Yang Y (2017). Long non-coding RNA SPRY4-IT1 promotes proliferation and invasion by acting as a ceRNA of miR-101-3p in colorectal cancer cells. Tumour Biol..

[96] Zhou M, Zhang XY, Yu X (2017). Overexpression of the long non-coding RNA SPRY4-IT1 promotes tumor cell proliferation and invasion by activating EZH2 in hepatocellular carcinoma. Biomed. Pharmacother..

[97] Peng W, Wu G, Fan H, Wu J, Feng J (2015). Long noncoding RNA SPRY4-IT1 predicts poor patient prognosis and promotes tumorigenesis in gastric cancer. Tumour Biol..

[98] Cui F (2016). Long noncoding RNA SPRY4-IT1 promotes esophageal squamous cell carcinoma cell proliferation, invasion, and epithelial-mesenchymal transition. Tumour Biol..

[99] Zhang E (2017). H3K27 acetylation activated-long non-coding RNA CCAT1 affects cell proliferation and migration by regulating SPRY4 and HOXB13 expression in esophageal squamous cell carcinoma. Nucleic Acids Res..

[100] Xin, Y., Li, Z., Zheng, H., Chan, M. T. V. & Ka Kei Wu, W. CCAT2: a novel oncogenic long non-coding RNA in human. *Cell Prolif*. **50**, e12342 (2017). 10.1111/cpr.12342 PMC6529071 28244168

[101] Ding J (2017). Long noncoding RNA CRNDE promotes colorectal cancer cell proliferation via epigenetically silencing DUSP5/CDKN1A expression. Cell Death Dis..

[102] Hu CE, Du PZ, Zhang HD, Huang GJ (2017). Long noncoding RNA CRNDE promotes proliferation of gastric cancer cells by targeting miR-145. Cell. Physiol. Biochem..

[103] Zhou X (2015). The interaction between MiR-141 and lncRNA-H19 in regulating cell proliferation and migration in gastric cancer. Cell. Physiol. Biochem..

[104] Ohtsuka M (2016). H19 noncoding RNA, an independent prognostic factor, regulates essential Rb-E2F and CDK8-beta-catenin signaling in colorectal cancer. EBioMedicine.

[105] Tsang WP (2010). Oncofetal H19-derived miR-675 regulates tumor suppressor RB in human colorectal cancer. Carcinogenesis.

[106] Zhao Y (2014). Role of long non-coding RNA HULC in cell proliferation, apoptosis and tumor metastasis of gastric cancer: a clinical and in vitro investigation. Oncol. Rep..

[107] Yang XJ, Huang CQ, Peng CW, Hou JX, Liu JY (2016). Long noncoding RNA HULC promotes colorectal carcinoma progression through epigenetically repressing NKD2 expression. Gene.

[108] Nie F (2017). Long noncoding RNA ZFAS1 promotes gastric cancer cells proliferation by epigenetically repressing KLF2 and NKD2 expression. Oncotarget.

[109] Han Y (2014). UCA1, a long non-coding RNA up-regulated in colorectal cancer influences cell proliferation, apoptosis and cell cycle distribution. Pathology.

[110] Zheng Q (2015). Aberrant expression of UCA1 in gastric cancer and its clinical significance. Clin. Transl. Oncol..

[111] Fu XL (2016). Analysis of long non-coding RNA expression profiles in pancreatic ductal adenocarcinoma. Sci. Rep..

[112] Liu XH (2014). LncRNA HOTAIR functions as a competing endogenous RNA to regulate HER2 expression by sponging miR-331-3p in gastric cancer. Mol. Cancer.

[113] Wang SH (2016). The lncRNA MALAT1 functions as a competing endogenous RNA to regulate MCL-1 expression by sponging miR-363-3p in gallbladder cancer. J. Cell. Mol. Med..

[114] Ji Q (2014). Long non-coding RNA MALAT1 promotes tumour growth and metastasis in colorectal cancer through binding to SFPQ and releasing oncogene PTBP2 from SFPQ/PTBP2 complex. Br. J. Cancer.

[115] Li C (2015). Progress and prospects of long noncoding RNAs (lncRNAs) in hepatocellular carcinoma. Cell. Physiol. Biochem..

[116] Liu YR (2015). Long noncoding RNAs in hepatocellular carcinoma: novel insights into their mechanism. World J. Hepatol..

[117] Bao H, Su H (2017). Long noncoding RNAs act as novel biomarkers for hepatocellular carcinoma: progress and prospects. Biomed. Res. Int..

[118] Wang X (2015). Silencing of long noncoding RNA MALAT1 by miR-101 and miR-217 inhibits proliferation, migration, and invasion of esophageal squamous cell carcinoma cells. J. Biol. Chem..

[119] Huang M (2017). Long noncoding RNA LINC00673 is activated by SP1 and exerts oncogenic properties by interacting with LSD1 and EZH2 in gastric cancer. Mol. Ther..

[120] Pan F (2014). A novel long non-coding RNA FOXCUT and mRNA FOXC1 pair promote progression and predict poor prognosis in esophageal squamous cell carcinoma. Int. J. Clin. Exp. Pathol..

[121] Wang CM (2014). Upregulation of the long non-coding RNA PlncRNA-1 promotes esophageal squamous carcinoma cell proliferation and correlates with advanced clinical stage. Dig. Dis. Sci..

[122] Zhang EB (2014). Long noncoding RNA ANRIL indicates a poor prognosis of gastric cancer and promotes tumor growth by epigenetically silencing of miR-99a/miR-449a. Oncotarget.

[123] Peng W, Fan H (2015). Long non-coding RNA PANDAR correlates with poor prognosis and promotes tumorigenesis in hepatocellular carcinoma. Biomed. Pharmacother..

[124] Li J (2017). PANDAR: a pivotal cancer-related long non-coding RNA in human cancers. Mol. Biosyst..

[125] Li Z, Shen J, Chan MT, Wu WK (2016). TUG1: a pivotal oncogenic long non-coding RNA of human cancers. Cell Prolif..

[126] Zhang E (2016). Increased expression of long noncoding RNA TUG1 predicts a poor prognosis of gastric cancer and regulates cell proliferation by epigenetically silencing of p57. Cell Death Dis..

[127] Zhai HY (2016). Overexpression of long non-coding RNA TUG1 promotes colon cancer progression. Med. Sci. Monit..

[128] Ma F (2017). Long non-coding RNA TUG1 promotes cell proliferation and metastasis by negatively regulating miR-300 in gallbladder carcinoma. Biomed. Pharmacother..

[129] Yang P (2017). The long noncoding RNA-ROR promotes the resistance of radiotherapy for human colorectal cancer cells by targeting the p53/miR-145 pathway. J. Gastroenterol. Hepatol..

[130] Zhou P, Sun L, Liu D, Liu C, Sun L (2016). Long non-coding RNA lincRNA-ROR promotes the progression of colon cancer and holds prognostic value by associating with miR-145. Pathol. Oncol. Res..

[131] Zang W (2015). Long noncoding RNA PEG10 regulates proliferation and invasion of esophageal cancer cells. Cancer Gene Ther..

[132] Xu MD (2014). Long non-coding RNA LSINCT5 predicts negative prognosis and exhibits oncogenic activity in gastric cancer. Medicine.

[133] Zhou J (2016). Knockdown of long noncoding RNA GHET1 inhibits cell proliferation and invasion of colorectal cancer. Oncol. Res..

[134] Chen WM (2015). Antisense long noncoding RNA HIF1A-AS2 Is upregulated in gastric cancer and associated with poor prognosis. Dig. Dis. Sci..

[135] Hu Y, Pan J, Wang Y, Li L, Huang Y (2015). Long noncoding RNA linc-UBC1 is negative prognostic factor and exhibits tumor pro-oncogenic activity in gastric cancer. Int. J. Clin. Exp. Pathol..

[136] Shi WH (2015). Upregulation of the long noncoding RNA PCAT-1 correlates with advanced clinical stage and poor prognosis in esophageal squamous carcinoma. Tumour Biol..

[137] Xie M (2015). Long noncoding RNA HOXA-AS2 promotes gastric cancer proliferation by epigenetically silencing P21/PLK3/DDIT3 expression. Oncotarget.

[138] Li W (2014). Increased levels of the long intergenic non-protein coding RNA POU3F3 promote DNA methylation in esophageal squamous cell carcinoma cells. Gastroenterology.

[139] Chen SX (2016). Upregulated expression of long noncoding RNA SNHG15 promotes cell proliferation and invasion through regulates MMP2/MMP9 in patients with GC. Tumour Biol..

[140] Lian Y (2016). The long noncoding RNA HOXA transcript at the distal tip promotes colorectal cancer growth partially via silencing of p21 expression. Tumour Biol..

[141] Zhang, F. et al. AFAP1-AS1: a novel oncogenic long non-coding RNA in human cancers. *Cell Prolif.* **51**, e12397 (2018). 10.1111/cpr.12397 PMC6528908 29057544

[142] Qi F (2017). Long noncoding AGAP2-AS1 is activated by SP1 and promotes cell proliferation and invasion in gastric cancer. J. Hematol. Oncol..

[143] Li, J. et al. Long non-coding RNA FOXP4-AS1 is an unfavourable prognostic factor and regulates proliferation and apoptosis in colorectal cancer. *Cell Prolif*. **50**, e12312 (2017). 10.1111/cpr.12312 PMC6529074 27790757

[144] Niu H (2016). Long non-coding RNA AK027294 involves in the process of proliferation, migration, and apoptosis of colorectal cancer cells. Tumour Biol..

[145] Wu X (2016). Long non-coding RNA ucoo2kmd.1 regulates CD44-dependent cell growth by competing for miR-211-3p in colorectal cancer. PLoS ONE.

[146] Xu Y (2016). Long noncoding RNA, tissue differentiation-inducing nonprotein coding RNA is upregulated and promotes development of esophageal squamous cell carcinoma. Dis. Esophagus.

[147] Yang L (2016). Upregulation of long non-coding RNA PRNCR1 in colorectal cancer promotes cell proliferation and cell cycle progression. Oncol. Rep..

[148] Shi D (2017). Silencing of long non-coding RNA SBDSP1 suppresses tumor growth and invasion in colorectal cancer. Biomed. Pharmacother..

[149] Han Y (2014). LEIGC long non-coding RNA acts as a tumor suppressor in gastric carcinoma by inhibiting the epithelial-to-mesenchymal transition. BMC Cancer.

[150] Sun M (2014). Decreased expression of long noncoding RNA GAS5 indicates a poor prognosis and promotes cell proliferation in gastric cancer. BMC Cancer.

[151] Yin D (2014). Long noncoding RNA GAS5 affects cell proliferation and predicts a poor prognosis in patients with colorectal cancer. Med. Oncol..

[152] Chang L (2016). Decreased expression of long non-coding RNA GAS5 indicates a poor prognosis and promotes cell proliferation and invasion in hepatocellular carcinoma by regulating vimentin. Mol. Med. Rep..

[153] Yan J (2014). MiR-148a regulates MEG3 in gastric cancer by targeting DNA methyltransferase 1. Med. Oncol..

[154] Peng W (2015). Long non-coding RNA MEG3 functions as a competing endogenous RNA to regulate gastric cancer progression. J. Exp. Clin. Cancer Res..

[155] Sun M (2014). Downregulated long noncoding RNA MEG3 is associated with poor prognosis and promotes cell proliferation in gastric cancer. Tumour Biol..

[156] Yin DD (2015). Decreased expression of long noncoding RNA MEG3 affects cell proliferation and predicts a poor prognosis in patients with colorectal cancer. Tumour Biol..

[157] Ding J (2015). Long non-coding RNA Loc554202 induces apoptosis in colorectal cancer cells via the caspase cleavage cascades. J. Exp. Clin. Cancer Res..

[158] Du T (2016). Decreased expression of long non-coding RNA WT1-AS promotes cell proliferation and invasion in gastric cancer. Biochim. Biophys. Acta.

[159] Li Y (2016). Decreased expression of LncRNA SLC25A25-AS1 promotes proliferation, chemoresistance, and EMT in colorectal cancer cells. Tumour Biol..

[160] Fei ZH (2016). Upregulated expression of long non-coding RNA LINC00982 regulates cell proliferation and its clinical relevance in patients with gastric cancer. Tumour Biol..

[161] Guo W (2016). Aberrant methylation-mediated downregulation of long noncoding RNA LOC100130476 correlates with malignant progression of esophageal squamous cell carcinoma. Dig. Liver Dis..

[162] Nie FQ (2016). Decreased long noncoding RNA MIR31HG is correlated with poor prognosis and contributes to cell proliferation in gastric cancer. Tumour Biol..

[163] Cao Y (2017). p53-inducible long non-coding RNA PICART1 mediates cancer cell proliferation and migration. Int. J. Oncol..

[164] Jiao C (2016). lncRNA-UCA1 enhances cell proliferation through functioning as a ceRNA of Sox4 in esophageal cancer. Oncol. Rep..

[165] Li JY, Ma X, Zhang CB (2014). Overexpression of long non-coding RNA UCA1 predicts a poor prognosis in patients with esophageal squamous cell carcinoma. Int. J. Clin. Exp. Pathol..

[166] Yu X, Zheng H, Chan MT, Wu WKK (2017). BANCR: a cancer-related long non-coding RNA. Am. J. Cancer Res..

[167] Shi Y (2015). Downregulated long noncoding RNA BANCR promotes the proliferation of colorectal cancer cells via downregualtion of p21 expression. PLoS ONE.

[168] Li L, Zhang L, Zhang Y, Zhou F (2015). Increased expression of LncRNA BANCR is associated with clinical progression and poor prognosis in gastric cancer. Biomed. Pharmacother..

[169] Yang X (2014). Long non-coding RNA HNF1A-AS1 regulates proliferation and migration in oesophageal adenocarcinoma cells. Gut.

[170] Dang Y (2015). Expression and clinical significance of long non-coding RNA HNF1A-AS1 in human gastric cancer. World J. Surg. Oncol..

