# Supplementary material for: Long non-coding RNAs: crucial regulators of gastrointestinal cancer cell proliferation
Source: Cell Death Discov. 2018 Apr 27;4:50. doi: 10.1038/s41420-018-0051-8 (PMC5919979; doi:10.1038/s41420-018-0051-8)
Supplement: Supplementary file 1 — Table S1 [file 41420_2018_51_MOESM1_ESM.docx]

**Table S1. Pro-proliferative lncRNAs are upregulated in certain digestive cancers, the molecular mechanisms and signaling pathways through which they act on specific targets and their pathophysiological functions besides promoting tumor growth.**

| LncRNA | Expression Level | Molecular Mechanisms | Downstream Target Genes | Signaling Pathway Involved | | Cancer type | Other Functions | References |
| --- | --- | --- | --- | --- | --- | --- | --- | --- |
| SPRY4-IT4 | upregulated | ceRNA for miR-101-3p |  |  | | CRC | EMT | ^95^ |
|  | upregulated | binds to EZH2 | E-cadherin$\downarrow$ |  | | HCC | EMT | ^96^ |
|  |  |  |  |  | | GC | prognosis, EMT | ^97^ |
|  |  |  |  |  | | ESCC | invasion, EMT | ^98^ |
| upat | upregulated | interferes with ubiquitination | UHRF1$\uparrow$; SCD1 and APRY4$\uparrow$ |  | | CRC | tumorigenesis, apoptosis | ^31^ |
| CCAT1 | upregulated |  | c-MYC$\uparrow$ |  | | CRC | invasion, migration | ^84^ |
|  |  |  |  | ERK/MAPK | | GC |  |  |
|  |  | ceRNA for let-7; miR-490-3p | HMGA2 and c-MYC$\uparrow$; CDK1$\uparrow$ |  | | HCC |  |  |
|  |  | binds to PRC2 and SUV39H; ceRNA for miR-7 | SPRY4$\downarrow$; HOXB13$\uparrow$ |  | | ESCC | migration, cell adhesion | ^99^ |
|  |  | ceRNA for miR-218-5p | Bmi1$\uparrow$ |  | | GBC | invasion | ^43, 84^ |
| ccat2 | upregulated |  | TCF7L2 and MYC$\uparrow$ | Wnt/β-catenin | | CRC | migration, microsatellite instability (MSI) | ^76, 100^ |
|  |  |  |  |  | | GC, HCC | migration, apoptosis | ^100^ |
|  |  |  | MYC$\uparrow$ |  | | ESCC | prognosis | ^100^ |
| CRNDE | upregulated | ceRNA for miR-181a-5p; ceRNA for miR-217; binds to EZH2 | cyclinD1 and Axin2$\uparrow$; TCF7L2$\uparrow$; DUSP5 and CDKN1A$\downarrow$ | Wnt/β-catenin | | CRC | apoptosis, invasion, migration | ^73, 74, 101^ |
|  |  | ceRNA for miR-145 | E2F3$\uparrow$ |  | | GC |  | ^102^ |
| H19 | upregulated | H19/miR-675 axis; ceRNA for miR-141 | RUNX1$\downarrow$; ZEB1, IGF1r and IGF2$\uparrow$ | Akt | | GC | tumorigenesis, invasion, migration, prognosis | ^51, 60, 103^ |
|  |  | CDK4 and cyclin D1$\downarrow$; CDKB$\downarrow$ | RB1-E2F1$\downarrow$; β-catenin$\downarrow$; RB$\downarrow$ | Wnt/β-catenin | | CRC | prognosis, methotrexate resistance | ^104, 105^ |
|  |  | inhibit miR-194-5p | AKT2$\uparrow$ |  | | GBC | EMT, invasion, metastasis | ^43^ |
| HULC | upregulated |  | EEF1E1 (p18)$\downarrow$ | p53 | | HCC | tumorigenesis, lipogenesis, EMT, invasion, migration | ^48^ |
|  |  |  |  |  | | GC | apoptosis, EMT, invasion, migration | ^106^ |
|  |  | binds to EZH2 | NKD2$\downarrow$ | | Wnt | CRC | apoptosis, invasion, migration, prognosis | ^107^ |
|  |  |  |  |  | | PC | prognosis | ^48^ |
| PVT1 | upregulated | binds to EZH2; ceRNA for miR-186; ceRNA for miR-152 | p15 and p16$\downarrow$; HIF-1α$\uparrow$; CD151 and FDF2$\uparrow$ |  | | GC | apoptosis, invasion, metastasis | ^82^ |
|  |  |  | c-MYC, FUBP1, EZH2 and NPM1$\uparrow$ | TGF-β | | CRC | apoptosis, invasion |  |
|  |  | stabilize and reduce degradation of NOP2 | NOP2$\uparrow$ | TGF-β | | HCC | prognosis |  |
|  |  |  | p21$\downarrow$ ZEB1 and Snail$\uparrow$ |  | | PC | EMT, migration |  |
| CSCA11 | upregulated | targets hnRNP-K | β-catenin and cyclinD1$\uparrow$ | Wnt/β-catenin | | CRC | metastasis, prognosis | ^75^ |
| ZFAS1 | upregulated | ceRNA for miR-590-3p | CDK1/cyclin B1 complex$\uparrow$ | p53 | | CRC | apoptosis | ^47^ |
|  |  | binds to EZH2 and LSD1 | KLF2 and NKD2$\downarrow$ |  | | GC | tumorigenesis, apoptosis, prognosis | ^108^ |
| UCA1 | upregulated |  |  |  | | CRC | apoptosis | ^109^ |
|  |  | binds to EZH2 | cyclin D1$\uparrow$ | Akt | | GC | prognosis, tumorigenesis | ^27, 110^ |
|  |  |  |  |  | | PDAC | invasion, prognosis | ^111^ |
| HOTAIR | upregulated | ceRNA for miR-331-3p | HER2$\uparrow$ |  | | GC | invasion, migration | ^112^ |
|  |  | induces hypermethylation of miR-130a promoter | miR-130a$\downarrow$ | MYC | | GBC | invasion | ^43^ |
| MALAT1 | upregulated | ceRNA for miR-363-3p | MCL-1$\uparrow$ | ERK/MAPK | | GBC | metastasis | ^68, 113^ |
|  |  | binds to SFPQ | PTBP2$\uparrow$ |  | | CRC | metastasis | ^114^ |
|  |  | changes distribution of SF2/ASF in nuclear speckles | SF2/ASF$\uparrow$ |  | | GC | differentiation | ^19^ |
|  |  |  |  |  | | HCC | prognosis, apoptosis, invasion, liver fibrosis | ^115, 116, 117^ |
|  |  | interacts with miR-101 and miR-217 | p21 and p27$\downarrow$; B-MYB$\uparrow$ | ATM/CHK2 | | ESCC | apoptosis, invasion, metastasis | ^92, 118^ |
| LINC00152 | upregulated | binds to EZH2; ceRNA to miR-18a-5p | p15 and p21$\downarrow$; THBS1$\uparrow$ |  | | GC | apoptosis, invasion, migration, EMT | ^56^ |
|  |  | ceRNA for miR-193a-3p | ERBB4$\uparrow$ |  | | CRC | apoptosis, chemoresistance |  |
|  |  |  | EpCAM$\uparrow$ | Akt/mTOR | | HCC |  |  |
|  |  |  |  | PI3K/Akt | | GBC | apoptosis, metastasis |  |
| linc00673 | upregulated | binds to EZH2 and LSD1 | KLF2 and LATS2$\uparrow$ |  | | GC | apoptosis, invasion | ^119^ |
| gclnc1 | upregulated | binds to WDR5 and KAT2A complex | SOD2$\uparrow$ |  | | GC | prognosis, invasion, metastasis, chemotherapy resistance | ^28^ |
| FOXCUT | upregulated | acts as RNA enhancer for FOXC1 | FOXC1$\uparrow$ |  | | ESCC | invasion, migration, prognosis | ^120^ |
| Plncrna-1(cbr3-as1) | upredulated |  |  |  | | ESCC | apoptosis, prognosis | ^121^ |
| anril(cdkn2b-as1) | upregulated | binds to PRC2 | p15 and p16$\downarrow$; miR-99a/miR-449a$\downarrow$; mTOR, CDK6 and E2F1$\uparrow$ | mTOR | | GC | prognosis | ^122^ |
|  |  |  | ki-67$\uparrow$; caspase-3$\downarrow$ |  | | GBC | apoptosis | ^43^ |
| KRT7-AS | upregulated | hybrids with KRT7 | KRT7$\uparrow$ |  | | GC | migration, prognosis | ^21^ |
| pandar (panda) | upregulated |  |  | p53 | | HCC | prognosis, invasion | ^123, 124^ |
|  |  |  | E-cadherin$\downarrow$; N-cadherin, vimentin, Snail and Twist$\uparrow$ |  | | CRC | apoptosis, EMT, invasion, migration | ^124^ |
|  |  |  |  |  | | GC | prognosis | ^124^ |
| CCHE1 | upregulated |  |  | ERK/MAPK | | HCC | apoptosis, prognosis | ^69^ |
| tug1 | upregulated | binds to PRC2 | KLF2$\downarrow$ |  | | HCC | tumorigenesis, apoptosis | ^125^ |
|  |  | binds to EZH2 | p15, p16, p21, p27 and p57$\downarrow$ |  | | GC | tumorigenesis, prognosis | ^126^ |
|  |  |  |  |  | | CRC | apoptosis, migration | ^127^ |
|  |  | binds to PRC2 |  |  | | ESCC | migration | ^125^ |
|  |  | ceRNA for miR-300 |  | TGF-β | | GBC | EMT, metastasis | ^128^ |
| linc-ror | upregulated | ceRNA for miR-145 |  | p53 | | CRC | apoptosis, invasion, migration, prognosis, radiotherapy resistance | ^49, 50, 129, 130^ |
|  |  |  |  | HIF-1α | | HCC | apoptosis, invasion, metastasis, prognosis | ^49^ |
| peg10 | upregulated |  |  |  | | EC | proliferation, cell cycle, apoptosis | ^131^ |
| lsinct5 | upregulated |  |  |  | | CRC, GC | proliferation, prognosis | ^132^ |
| ghet1 | upregulated | increases interaction between IGF2BP1 and c-MYC mRNA | c-MYC$\uparrow$ | MYC | | GC | prognosis | ^88^ |
|  |  |  |  |  | | CRC | EMT, invasion, migration | ^133^ |
| hif1a-as2 | upregulated |  |  |  | | GC | prognosis | ^134^ |
| linc-ubc1 | upregulated |  |  |  | | GC | invasion, prognosis | ^135^ |
| pcat-1 | upregulated |  |  |  | | ESCC | invasion, prognosis | ^136^ |
| hoxa-as2 | upregulated | binds to EZH2 | p21, PLK3 and DDIT3$\downarrow$ |  | | GC | prognosis, apoptosis | ^137^ |
| Linc-pou3f3 | upregulated | promotes T-reg differentiation | TGF-β, SMAD2 and SMAD3$\uparrow$ | TGF-β | | GC |  | ^89^ |
|  |  | binds to EZH2 | POU3F3$\downarrow$ |  | | ESCC |  | ^138^ |
| snhg15 | upregulated |  | MMP2/MMP9 |  | | GC | apoptosis, invasion, prognosis | ^139^ |
| HOTTIP | upredulated |  | p21$\downarrow$ |  | | CRC | apoptosis | ^140^ |
| afap1-as1 | upregulated |  | PTEN$\uparrow$; p-AKT$\downarrow$; Bcl-2$\downarrow$; cleaved PARP, caspase-3/9 and Bax$\uparrow$ | PTEN/Akt | | GC | apoptosis | ^141^ |
|  |  |  |  |  | | CRC | invasion, migration, EMT |  |
|  |  |  | Bax$\uparrow$; cyclin D1$\downarrow$ | RhoA/Rac2 | | HCC | apoptosis, invasion, migration |  |
|  |  |  | G2/M arrest |  | | PDAC | invasion, migration, EMT |  |
|  |  |  |  |  | | EC | apoptosis, invasion, migration |  |
| agap2-as1 | upregulated | binds to EZH2 and LSD1 | p21 and E-cadherin$\downarrow$ |  | | GC | invasion, migration | ^142^ |
| foxp4-as1 | upredulated |  | p15, p21, p27, KLF2$\downarrow$ |  | | CRC | apoptosis, prognosis, tumorigenesis | ^143^ |
| ak027294 | upregulated |  | Bcl-2$\uparrow$; caspase-3/9$\downarrow$; MMP9/12$\uparrow$ |  | | CRC | migration, apoptosis | ^144^ |
| gacAt3 | upregulated |  |  | IL6/STAT3 | | GC |  | ^94^ |
| LNCRNA-uc002kmd.1(GAPLINC) | upregulated | ceRNA for miR-211-3P | CD44$\uparrow$ |  | | GC, CRC | migration | ^145^ |
| tincr | upregulated |  |  |  | | ESCC | invasion, migration | ^146^ |
| prncr1 | upregulated |  |  |  | | CRC |  | ^147^ |
| PCAT-1 | upregulated |  | c-MYC$\uparrow$ |  | | CRC | apoptosis | ^83^ |
|  |  |  |  |  | | ESCC | invasion, prognosis | ^136^ |
| SBDSP1 | upregulated |  | p21 and cyclin D1$\uparrow$; phosphorylated Akt, ERK1/2, STAT3$\uparrow$ |  | | CRC | tumorigenesis, invasion, migration | ^148^ |
