# Supplementary material for: Long non-coding RNAs: crucial regulators of gastrointestinal cancer cell proliferation
Source: Cell Death Discov. 2018 Apr 27;4:50. doi: 10.1038/s41420-018-0051-8 (PMC5919979; doi:10.1038/s41420-018-0051-8)
Supplement: Supplementary file 2 — Table S2 [file 41420_2018_51_MOESM2_ESM.docx]

**Table S2. Anti-proliferative lncRNAs are downregulated in certain digestive cancers, the molecular mechanisms and signaling pathways through which they act on specific targets and their pathophysiological functions besides inhibiting tumor growth.**

| LncRNA | Expression Level | Molecular Mechanisms | Downstream Target Genes | Signaling Pathway Involved | Cancer Category | Other Functions | References |
| --- | --- | --- | --- | --- | --- | --- | --- |
| loc285194 (TUSC7) | downregulated | forms reciprocal repression feedback loop with miR-211 | miR-211$\downarrow$ | p53 | CRC |  | ^35^ |
|  |  | forms reciprocal repression feedback loop with miR-23b | miR-23b$\downarrow$ | p53 | GC | prognosis, invasion | ^36^ |
| leigc | downregulated |  |  |  | GC | invasion, EMT, 5-FU sensitivity | ^149^ |
| gas5 | downregulated |  | E2F1 and cyclin D1$\downarrow$; p21$\uparrow$ |  | GC | prognosis, apoptosis | ^150, 151^ |
|  |  |  | Vimentin$\uparrow$; E-cadherin$\downarrow$ |  | HCC | invasion, apoptosis, EMT | ^152^ |
| lincrna-p21 | downregulated | inhibits mRNA translation of JunB and CTNNB1 | JunB and β-catenin$\downarrow$ | Wnt/β-catenin | CRC | tumorigenesis | ^39, 40^ |
|  |  |  |  | ER stress | HCC | apoptosis, liver fibrogenesis | ^39^ |
| meg3 | downregulated |  | p53$\uparrow$ | p53 | HCC | apoptosis, prognosis | ^42, 153^ |
|  |  |  | p53 and caspase-3$\uparrow$; cyclin D1$\downarrow$ | p53 | GBC | apoptosis | ^43^ |
|  |  | ceRNA for miR-181a | Bcl-2$\uparrow$ | p53 | GC | apoptosis, prognosis, invasion | ^154, 155, 156^ |
|  |  |  |  |  | CRC |  | ^157^ |
|  |  |  | GRP78, IRE1, PERK, ATF6, CHOP and cleaved-caspase3$\uparrow$ | ER stress | ESCC | apoptosis | ^93^ |
| lncrna-let | downregulated | decreases NF90 stability | HIF-1α$\downarrow$ | HIF-1α | HCC, GBC, GC | apoptosis, migration | ^43, 45, 46^ |
|  |  |  | p53$\uparrow$ | p53 | ESCC | apoptosis, invasion, metastasis | ^44^ |
| loc554202 | downregulated | activates specific caspase cleavage cascades | Bax, cleavage caspase-3 and 9$\uparrow$; bcl-2$\downarrow$ |  | CRC | apoptosis | ^158^ |
| fer1l4 | downregulated | ceRNA for miR-106a-5p | PTEN$\uparrow$ |  | GC |  | ^63^ |
| wt1-as | downregulated |  | phosphorylated ERK$\uparrow$ |  | GC | migration, invasion | ^159^ |
| slc25a25-as1 | downredulated |  |  |  | CRC | chemoresistance, EMT | ^160^ |
| LINC00982 |  |  | p15 and p21$\uparrow$ |  | GC | prognosis | ^161^ |
| loc100130476 | downregulated |  |  |  | ESCC | invasion, prognosis | ^162^ |
| mir31hg | downregulated |  | E2F1$\downarrow$; p21$\uparrow$ |  | GC | prognosis | ^163^ |
| PICART1 | downregulated |  | AKT, β-catenin, cyclin D1 and c-MYC$\downarrow$; p21Waf/cip1$\uparrow$ | Akt/GSK3β | CRC | invasion, migration | ^164^ |
| rp11-708h21.4 | downregulated |  | cyclin D1$\downarrow$; p27$\uparrow$ | Akt/mTOR | CRC | apoptosis, invasion, migration, prognosis, 5-FU sensitivity | ^66^ |
