# Supplementary material for: Long non-coding RNAs: crucial regulators of gastrointestinal cancer cell proliferation
Source: Cell Death Discov. 2018 Apr 27;4:50. doi: 10.1038/s41420-018-0051-8 (PMC5919979; doi:10.1038/s41420-018-0051-8)
Supplement: Supplementary file 3 — Table S3 [file 41420_2018_51_MOESM3_ESM.docx]

**Table S3. LncRNAs with reverse expression levels in the same or different digestive cancers, the molecular mechanisms and signaling pathways through which they act on specific targets and their pathophysiological functions besides influencing tumor growth.**

| LncRNA | Expression Level | Molecular Mechanisms | Downstream Target Genes | Signaling Pathway Involved | Cancer Category | Other Functions | References |
| --- | --- | --- | --- | --- | --- | --- | --- |
| uca1 | upregulated | ceRNA for miR-204 | Sox4$\uparrow$ |  | ESCC | prognosis, invasion, migration | ^165, 166^ |
|  | downregulated |  | c-MYC and β-catenin$\downarrow$ | Wnt/β-catenin | ESCC | invasion, migration | ^78^ |
| BANCR | upregulated |  |  | MEK/ERK | CRC | EMT, migration | ^167^ |
|  | downregulated |  | p21$\uparrow$ |  | CRC | apoptosis, | ^167, 168^ |
|  | upregulated | regulates miR-9 | NF-KB1$\uparrow$ |  | GC | apoptosis, prognosis | ^167, 169^ |
| hnf1a-as1 | upregulated |  | H19$\uparrow$ |  | EAC | anchorage-independent growyh, invasion, migration | ^170^ |
|  | downregulated |  |  |  | GC |  | ^171^ |
